# Novel prospects for scarless wound healing: The roles of myofibroblasts and adipocytes

**DOI:** 10.1111/jcmm.17535

**Published:** 2022-09-15

**Authors:** Ying‐Ying Teng, Ming‐Li Zou, Xiao‐Jin Zhou, Jun‐Jie Wu, Si‐Yu Liu, Zheng‐Dong Yuan, Yuan Jia, Kai‐Wen Zhang, Xia Li, Jun‐Xing Ye, Feng‐Lai Yuan

**Affiliations:** ^1^ Institute of Integrated Chinese and Western Medicine The Hospital Affiliated to Jiangnan University Wuxi China; ^2^ Wuxi Clinical Medicine School of Integrated Chinese and Western Medicine Nanjing University of Chinese Medicine Wuxi China

**Keywords:** adipocytes, myofibroblast, reprogramming, scarless, wound healing

## Abstract

Disturbances or defects in the process of wound repair can disrupt the delicate balance of cells and molecules necessary for complete wound healing, thus leading to chronic wounds or fibrotic scars. Myofibroblasts are one of the most important cells involved in fibrotic scars, and reprogramming provides a potential avenue to increase myofibroblast clearance. Although myofibroblasts have long been recognized as terminally differentiated cells, recent studies have shown that myofibroblasts have the capacity to be reprogrammed into adipocytes. This review intends to summarize the potential of reprogramming myofibroblasts into adipocytes. We will discuss myofibroblast lineage tracing, as well as the known mechanisms underlying adipocyte regeneration from myofibroblasts. In addition, we investigated different changes in myofibroblast gene expression, transcriptional regulators, signalling pathways and epigenetic regulators during skin wound healing. In the future, myofibroblast reprogramming in wound healing will be better understood and appreciated, which may provide new ideas for the treatment of scarless wound healing.

## 1 INTRODUCTION

Various types of wounds, including traumatic wounds, acute postsurgical wounds and burns, have a strong influence on patients and society.^1^ Effective wound healing involves successive phases of inflammation, proliferation and reconstruction after initial haemostasis,^2^, ^3^ including complex coordination between resident and recruited cells. Disorders or defects in these processes, however, can disrupt the delicate balance of cell types and molecules necessary for complete wound healing, thus leading to chronic trauma and fibrotic scarring.^4^, ^5^ Furthermore, one of the most significant cells involved in fibrotic scarring are myofibroblasts, which can be activated by transforming growth factor‐beta (TGF‐β) and deposit amounts of extracellular matrix (ECM), such as collagens 1 and 3.^6^, ^7^ Myofibroblasts are special cells with characteristics of both fibroblasts and smooth muscle cells, thereby expressing alpha‐smooth muscle actin (α‐SMA) and generating increased contractile forces. Myofibroblasts are terminal cells differentiated from fibroblasts, which have contractile function and active secretion function. They participate in the process of wound contraction and tissue fibrosis. When the function is completed, it is resolved by apoptosis.^8^ However, myofibroblast accumulation, excessive ECM deposition and stronger contractions eventually lead to scar formation. Therefore, it is necessary to clearly understand the birth, development, function and fate of myofibroblasts in an effort to prevent and treat scar formation.

Myofibroblasts have long been known to be terminally differentiated cells. Recent study has shown that myofibroblasts, cells thought to be non‐adipogenic, can be reprogrammed to adipocytes during wound repair, thus demonstrating the potential of myofibroblast transdifferentiation.^9^ Adipocytes are able to produce fatty acids and hormones to regulate gluconeogenesis and inflammation.^10^, ^11^ The functions of adipocytes have sparked speculation that adipocytes might actively participate in the wound healing process,^12^ vis‐a‐vis secretion of growth factors, angiogenesis and production of collagen type VI. Hence, it is important to study the possibility of adipocyte regeneration from myofibroblasts.

Thus, we conducted a review of the current literature and discussed myofibroblast lineage tracing and the mechanisms known to underlie adipocyte regeneration from myofibroblasts. Moreover, we have investigated different changes in myofibroblast gene expression, transcriptional regulators, signalling pathways and epigenetic regulators during cutaneous wound healing. Furthermore, we believe that the information we have collected, reviewed and summarized will contribute to a better understanding of the mechanisms underlying myofibroblast reprogramming in wound healing and the development of new treatment strategies.

## 2 MYOFIBROBLASTS LINEAGE

Advances in genetic fate tracing have made it possible to identify, characterize and fate map progenitor and multipotent stem cells in homeostatic and disease conditions. Myofibroblasts that are responsible for scarring in skin fibrosis have been confirmed to be derived from dermal fibroblasts in situ. Murine skin fibroblasts have been shown to originate from two different embryonic lineages (the upper dermis, which is composed of leucine‐rich repeats and immunoglobulin‐like domains protein1‐positive [LRIG1+] fibroblasts and the lower dermis, which is composed of delta‐like homologue 1‐positive [DLK1+] fibroblasts).^13^ Driskell et al.^14^ verified that the upper dermis is papillary and responsible to re‐epithelization and hair follicle formation, whereas the lower dermis is composed of reticular fibroblasts, which can deposit amounts of ECM and contribute to wound repair after injury. These fibroblasts always infiltrate the wound bed first with α‐SMA expression, a myofibroblast phenotype.^15^

**FIGURE 1:**
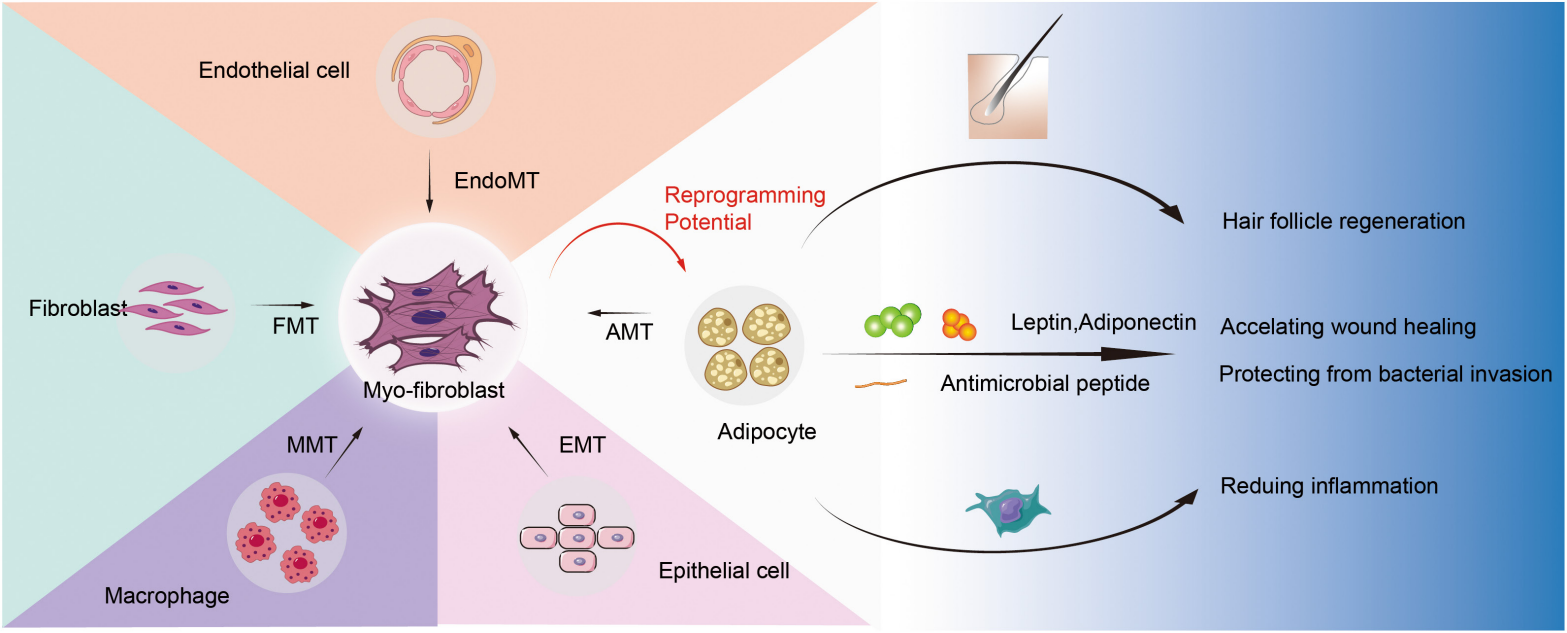
Myofibroblasts can be transformed from fibroblasts, endothelial cells, epithelial cells, adipocytes and macrophages, and are considered to be terminally differentiated cells. Myofibroblasts have the potential to reprogram into fat cells. AMT, adipocyte‐to‐myofibroblast transition; EMyT, epithelial‐to‐myofibroblast transition; End‐MyoT, endothelial‐to‐myofibroblast transition; FMT, fibroblast‐to‐myofibroblast transition; MMT, macrophage‐to‐myofibroblast transition. Adipocytes play an important role in the wound healing process.

Some studies have reported that apart from the transition from fibroblasts‐to‐myofibroblasts, myofibroblasts in skin fibrosis also originate from other types of cells, including endothelial‐to‐myofibroblast transition (End‐MyoT),^16^ epithelial‐to‐myofibroblast transition (EMyT)^14^, ^17^ and adipocyte‐to‐myofibroblast transition (AMT).^18^ Interestingly, another recently described mechanism involves macrophage‐to‐myofibroblast transition (MMT),^19^ which might play an even more important role in active fibrotic lesions, such as kidney disease and myocardial infarction. Zeisberg et al.^20^ demonstrated the endothelial origin of myofibroblasts by demonstrating the co‐expression of CD31 in endothelial cells and the myofibroblast marker, αSMA, in a mouse fibrosis model. In fact, the important role of endothelial cells and their contribution to the myofibroblast translation in skin fibrosis is largely unknown and controversial^21^ (Figure 1).

Since myofibroblasts are abundantly sourced and only largely found in pathologically repaired tissues, people have long regarded them as the terminal form of cell differentiation thus ignoring their possibility of reprogramming to other cells. Coincidently, Plikus et al.^9^ noticed that the adult mice that healed without scarring would form new adipocytes within the wound area around new hair follicles. This triggered their thinking about the lost myofibroblasts and the new adipocytes. Would it be possible that the disappearance of myofibroblasts is more than just their own apoptosis? Is there any possibility that the new adipocytes are derived from myofibroblasts? Subsequent studies by them verified that myofibroblasts can be reprogrammed into adipose precursor cells, uncovering the reprogramming of myofibroblasts. Interestingly, a recent report demonstrated that myofibroblasts can revert to lipofibroblast‐like cells in the resolution of lung fibrosis. Following the resolution of fibrosis, the Acta2 gene was significantly reduced by 4.7 times and the adipogenic differentiation‐related gene Adrp increased. At the same time, PPARg, a main regulator of adipogenesis, also increased clearly. The increase in the number of cells staining positive for neutral lipid staining also supported this view.^22^ The BMP and PPARg signalling pathway participate in reversing the reprogram of adipocytes into myofibroblasts by activating genes related to adipogenesis, adipocyte differentiation and cholesterol metabolism.^23^ Given that both types of cells are mesodermal, the reprogramming of myofibroblasts into adipocytes might not be that surprising.^24^ Next, a single‐cell analysis revealed that a subpopulation of myofibroblasts originated from the bone marrow. Bone marrow transplantation and lineage tracking experiments proved that this circulating myeloid cells produced myofibroblasts and regenerated fat cells. This directly relevant work verified the differentiation process from myeloid cells to myofibroblasts and then to adipocytes.^25^ Therefore, regenerating myofibroblast into adipocytes might be a promising treatment for skin fibrosis.

## 3 EXPERIMENTAL MYOFIBROBLAST‐TO‐ADIPOCYTE REPROGRAMMING

Myofibroblasts are the main type in scar tissue and the persistence of myofibroblasts after injury is the leading cause of scarring in mammals. Brewer et al.^26^ reduced the in vitro formation of myofibroblasts by inhibiting Yap activity, further promoting scar‐free healing. While it is recognized that inhibiting the transition of fibroblasts‐to‐myofibroblasts has the potential to prevent fibrosis in response to injury and prevent progression of established fibrotic processes, the transition is unlikely to promote complete elimination of fibrosis. In contrast, promoting myofibroblast clearance holds promise. Because the resolution of myofibroblasts in fibrosis is limited by the anti‐apoptotic ability, dedifferentiation and reprogramming provide potential avenues to promote myofibroblast clearance. New insights into the function and composition of adipose tissue have fuelled speculation about its regenerative potential over the past few years. In this regard, adipo‐derived stem cells (ASCs) and autologous fat grafting have been used to treat a variety of diseases, including burn wound, ulcers and breast reconstruction after mastectomy.^27^, ^28^, ^29^ Adipocytes play an important role in the fibrosis process. The process of skin fibrosis in systemic sclerosis is often accompanied by a continuous loss of dermal adipose tissue, a decrease in the size and number of fat cells and subsequent increase in high‐density collagen production.^30^ Lineage tracing and in vitro differentiation experiments in mice showed that fat cells in the skin transformed from fat cells to myofibroblasts by AMT, and the expression of myofibroblast markers increased and led to fibrotic phenotypes. Furthermore, adiponectin‐expressing cells acquire actin alpha 2 (ACTA2) expression and lose the adipogenic gene signature,^18^ which is thought to prove the translation from AMT. Additionally, adiponectin affects the progression of fibrosis in scleroderma patients who are subjected to the loss of subcutaneous adipose tissue.^31^ Moreover, increased studies have reported the effect of adipocytes on wound healing. Schmidt and Horsley demonstrated that adipocyte precursor cells proliferated after injury, and mature adipocytes refilled skin wounds after inflammation, which is essential for wound healing. The proliferative phase of skin wound healing requires fat cells to guide the function of fibroblasts.^10^ It has previously been demonstrated that adipose cells regulate α‐SMA expression which can increase the tension of the skin around the wound and promote contraction.^32^, ^33^, ^34^ Furthermore, adipocytes can promote the proper healing of the skin wound through paracrine methods. The secreted adipokines can also reduce skin fibrosis. Adiponectin, one of the important adipokines, can affect the re‐epithelialization progress of wounds, and wound healing in mice lacking adiponectin is significantly delayed.^35^ Leptin, which is also secreted by adipocytes, possesses the ability to stimulate the proliferation of keratinocytes and fibroblasts, epithelialization and collagen production, leading to improve skin regeneration and shorten the time of wound healing.^36^, ^37^, ^38^ Noticeably, in transgenic mice with leptin receptor deficiency, anagen was found to be delayed which suggested that adipocytes could be an essential important anagen activator in the second hair cycle and may promote hair growth.^39^ In addition, a recent study has affirmed the importance of adipocytes in the local immune response to bacterial infections.^40^ As *Staphylococcus aureus* infects the skin, the rapidly increasing adipocytes begin to secrete large amounts of antibacterial peptides to kill bacteria, which protect the skin from bacterial invasion. Shook et al.^41^ used gene‐deletion mice to find that fat cells in mouse skin could reduce inflammation by releasing FAs to recruit macrophages; further single‐cell sequencing results showed that fat cells at the site of injury would differentiate into myofibroblasts to speed up wound repair. These data emphasize the interplay between adipocytes and myofibroblasts. It is this innovative view that attracts our sights on the reprogramming of myofibroblasts into adipocytes for the theory of the prevention and treatment of hypertrophic scars (Figure 1).

Indeed, myofibroblast dedifferentiation has been reported in response to multiple effector molecules. Mitogens (serum and fibroblast growth factors [FGFs]), lipid mediators (prostaglandin E2 [PGE2] and prostaglandin I2 [PGI2]) and Eukaryotic initiation factor 6 have been proven to promote the dedifferentiation process of myofibroblasts.^42^, ^43^ However, whether dedifferentiation with these different effector molecules produces common or different cellular phenotypes, and whether reprogramming phenomena appear during dedifferentiation, have not been previously studied.

**FIGURE 2:**
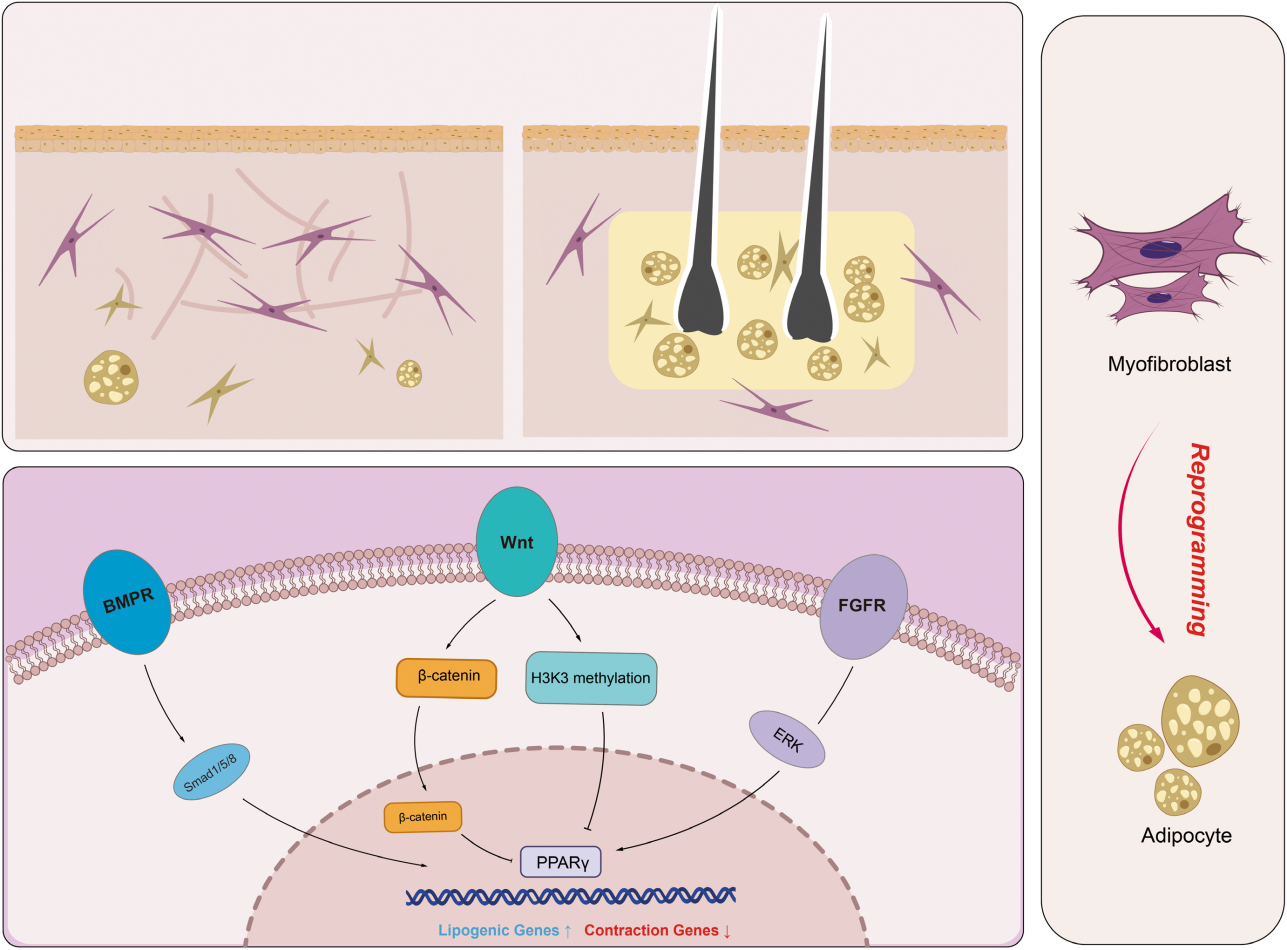
Thickness of the adipocyte layer increases dramatically where hair follicles exist, thus hair follicles play an important role in myofibroblasts into adipocytes and have potential for scar treatment. Mechanisms underlying myofibroblast‐to‐adipocyte reprogramming.

In contrast, a number of studies have focused on the role of dermal adipose tissue in skin regeneration over the past few years. Notably, adipocyte precursor cells and mature adipocytes are involved in a variety of regenerative and pathophysiologic processes in the skin, including hair follicle (HF) regeneration and fibroblast regeneration after injury.^44^ An emerging theme that has attracted attention involves the influence of hair follicles on the myofibroblast reduction and maturation of adipocyte lineage cells (Figure 2). A well‐known finding is the relationship between the HF growth cycle and oscillations in the thickness of the dermal adipocyte layer.^45^ When HF penetrates deep into the dermal adipocyte layer during the anagen phase of the cycle, the thickness of the adipocyte layer increases dramatically at the same time, which reflects an increase in lipogenesis and the volume of individual adipocytes. It has also been shown that activation of hair cycling by hair plucking or activated expression of Shh can induce skin lipogenesis. Rivera‐Gonzalez et al.^46^ revealed that PI3K/Akt2 played a role in fat proliferation as a direct target for Pdgfa through a dynamic lipogenic procedure that occurs during hair growth. Zhang et al.^47^ also demonstrated that new adipogenesis in the dermis begins during the middle stages of HF formation, continues throughout anagen and stops during catagen when the HFs are destroyed. This finding has tightened the link between HFs and skin adipogenesis. New fat cells do not form in the hairless parts of the wound, but only develop around new hair follicles. Zhang et al.^48^ defined its structure as a unique fat bank by isolating mature dermal fat cells and performing characterization detection and discovered the long‐term interaction between hair follicles and fat cells by using a large number of rodent models, which emphasized the important role of mature dermal fat cells in mouse hair circulation and skin wound healing.

Furthermore, the regenerated hair follicle can transform the surrounding myofibroblasts into fat cells by transmitting bone morphogenetic protein (BMP) signals, which promotes wound regeneration instead of scarring. Kulessa et al.^49^ confirmed that mature hair follicles strongly express BMP2 and BMP4, which may have an important regulatory effect on the reprogram of myofibroblasts into adipocytes. In the dermal cells next to the regenerating hair follicle, the expression of BMP signal is active, and the expression of pSMAD1/5/8, signature of BMP activation, is significantly up‐regulated. Plikus et al.^9^ provided further support that the fate of myofibroblasts into fat cells is able to respond to BMP signals from HFs. Therefore, combining WNT, FGF and BMP treatments to first stimulate hair follicle regeneration and then induce the reprogramming of scar‐formed myofibroblasts into adipocytes is a two‐stage scarless wound healing strategy. While other sources of signalling, such as the intercalation‐derived signal, platelet‐derived growth factor subunit A (Pdgfa), can also regulate proliferation of adipocyte precursor cells, future studies focusing on the source of signalling and how HFs affect the myofibroblasts will be essential to decipher the code of fat regeneration and regulation in the skin.

## 4 MECHANISMS UNDERLYING MYOFIBROBLAST‐TO‐ADIPOCYTE REPROGRAMMING

### 4.1 Gene expression

Mechanistic insight into the gene expression dynamics of myofibroblasts after injury has been gleaned through years of study on the transcriptome. Picelli et al.^50^ performed SMART‐seq2 on extracted, viable, uncultured FACS‐sorted tdTomatohi myofibroblast RNAs in mice at four time points after injury (Days 12, 15, 21 and 26). Picelli et al. identified 4120 transcripts that exhibited statistically significant differences in expression at all four time points using inferential statistical analysis with the two‐step regression model algorithm, maSigPro.^51^ Among the differentially expressed genes, the number of cell cycle regulators enriched at late time points after wounding decreased significantly. The genes associated with myofibroblast contractility were decreased on Day 15, which coincides with the period of new HF formation and is consistent with the active contractile state of myofibroblast closure in the late stage of wound healing. The enriched ECM and the number of secretory/signalling pathway genes increased significantly during wound adipogenesis on Days 21 and Day 26 when ZFP423nd pCEBPβ‐expressing dermal progenitor cells appeared, along with new HFs and new adipocyte maturation. With respect to the changes in gene expression in myofibroblasts after injury, fat formation occurs when genes that promote wound contraction are reduced. Then, the consistency of gene expression changes could predict the correlation and the possible relationship between myofibroblast reduction and adipocyte formation.

### 4.2 Transcriptional regulators

In the past decade, an abundance of transcriptional regulators that promote adipocyte recruitment and activation have been identified and extensively studied. Few studies, however, have reported the relationship between the changes in these regulatory factors and fat formation in the process of wound healing. Plikus et al.^9^ revealed that in the early stage of wound healing without adipogenesis, inhibitors of adipogenesis, such as Nr2f6 and E2f4, are highly expressed. When fat regeneration begins in the late stage of wound repair, several adipogenic activators, including Zfp423, Crebl2, Stat5b and Klf15, are highly up‐regulated, while Sox9/11, Runx1/2, Fhl2 and Pitx1, the transcriptional regulators of the chondrocyte line and osteoblast cell line, are down‐regulated. The signal transducer and activator of transcription 5 (STAT5) transcription factor,^52^ which can promote the transformation of non‐precursor fibroblasts into functional mature fat cells by C/EBPs and PPARs, was observed to be more highly expressed on Day 21 when HFs begin to regenerate.

### 4.3 Signalling pathways

Changes in transcriptional regulators are often accompanied by significant changes in major signalling pathways associated with adipose spectrum regulation. BMP‐4 is one of the key factors in reprogramming myofibroblasts into adipocytes. Overexpression of BMP (bone morphogenetic protein) antagonists and loss of BMP receptors impair adipogenesis. Another study reviewed the mechanism by which adipose precursor cells transform into adipocytes, indicating that an increase in BMP‐4 signalling increased the adipogenic potential of adipose precursor cells.^53^ At the same time, maintaining the BMP signalling pathway throughout the generative process can induce the stabilization of the human or murine adipocyte phenotype. BMP, which has been shown to promote angiogenesis,^54^ may also promote the formation of adipose tissue, a process that also requires new angiogenesis.

In contrast, peroxisome proliferation‐activated receptor γ (PPARγ), a member of the nuclear hormone receptor ligand‐activated transcription factor superfamily, is considered to be a master transcriptional regulator of adipocyte differentiation. Recent studies have shown that osteogenic differentiation inducers, such as Wnt, inhibit PPARγ activation through multiple mechanisms.^55^ Among the osteogenic induces, the canonical Wnt/β‐catenin pathway inhibits PPARγ mRNA expression, while the non‐canonical Wnt pathway activates histone methyltransferase, which inhibits PPARγ activation through histone H3 lysine 9 (H3K9) methylation of its target genes. It has recently been reported that Wnt5a may be a trigger for the dedifferentiation of adipocytes into adipocyte‐derived fibroblasts in the tumour microenvironment of pancreatic cancer.^56^ Wnt family‐assigned molecules exert adipogenic effects on adipocytes, while other molecules, such as Wnt10b or Wnt3a, have anti‐adipogenic effects. This ability may depend on different extracellular microenvironments, and it is interesting to determine that lipogenesis may be influenced by microenvironmental influences.

Moreover, the fibroblast growth factor (FGF) pathway exhibits different gene expression patterns in the late phase. FGF10 has previously been shown to be able to stimulate the proliferative expansion of adipose progenitor cells.^57^ Among other pathways, previous reports suggest that reduced insulin‐like growth factor 2 (IGF2) levels are associated with increased fat deposition and incident obesity. In addition, it has been shown that IGF2 inactivation and Myod are critical for the fate selection of brown fat and skeletal muscle cells^58^ (Figure 2).

### 4.4 Epigenetic regulators

Interestingly, some phenotypes of organisms are also altered in the absence of alterations in the DNA sequence.^59^ We have listed the key epigenomic regulators during myofibroblast reprogramming and adipogenesis in Table 1, including but not limited to the following factors: histone methyltransferases/demethylases; histone acetylases/deacetylases; epigenomic readouts; chromatin remodelling factors; DNA methylases/demethylases and microRNA. It is apparent that the function and mechanism underlying the regulation of some epigenetic enzymes are not clear, thus the role that epigenetic enzymes play in adipocyte formation requires further study and validation.

**TABLE 1:** Key epigenomic regulators during myofibroblast reprogramming and adipogenesis

Epigenomic regulators			Function	Refs.
Lysine methyltransferases	Mammalian Set1‐like H3K4 methyltransferases	MLL3/MLL4	Acceleration	[63, 64]
	H3K9 methyltransferases	G9a/Setdb1	Inhibition	[65]
	Dominant H3K27 trimethyltransferase Polycomb repressive complex 2 (PRC2)	Ezh2	Acceleration	[66]
Lysine demethylases	H3K4 and H3K9 demethylase	Lsd1	Acceleration	[67]
	H3K4 demethylases	Kdm5	Unclear in vivo	[68]
Arginine methyltransferases	Protein arginine methyltransferases (PRMTs)	Carm1	Acceleration	[69]
	PRMTs	Prmt5	Unclear	[70, 71]
Histone acetyltransferases.	Acetylation on H3K9 (H3K9ac)	Gcn5/PCAF	Acceleration	[72]
	Acetylation on H3K18 (H3K18ac) and H3K27 (H3K27ac)	CBP/p300	Acceleration	[73]
Histone deacetylases and sirtuins	Histone deacetylases (HDACs)	Hdac1/Hdac2	Acceleration	[74]
		Hdac9	Inhibition	[75]
	Sirtuins	Sirt1/Sirt2	Inhibition	[76, 77, 78]
		Sirt6/Sirt7	Acceleration	[79, 80]
Epigenomic reader	Bromodomain and extraterminal domain (BET) proteins	Brd4	Acceleration	[81]
Chromatin remodelling complex		SWI/SNF	Acceleration	[82]
microRNAs		miR‐378	Inhibition	[83]
		miR‐33	Acceleration	[84]

## 5 THERAPEUTIC POTENTIAL OF MYOFIBROBLAST‐TO‐ADIPOCYTE REPROGRAMMING

The heightened proliferation of myofibroblasts in the late stage of wound healing contributes to excessive collagen deposition, which greatly reduces the quality of wound healing. Local pathologic scars also bring numerous side effects. Depleting myofibroblasts during wound healing is a well‐established anti‐scarring strategy, but the potential of myofibroblast reprogramming is neglected, which was long considered to be terminally differentiated cells. Plikus et al.^9^ delved into the phenomenon that new adipocytes always form around new HFs and in 2017 reported that these newly formed adipocytes originate from myofibroblasts. That striking discovery provided us with new ideas for the prevention and treatment of pathologic scars. The Plikus et al.^9^ further explored the molecular regulatory mechanism by which hBMP4 mediates adipogenic transformation of human scar fibroblasts in vitro. After pretreatment of keloid fibroblasts with hBMP4, upregulation of adipogenic genes (ZNF423, AdipoQ, adipokine, PPARG2 and FABP4) occurred accompanied by adipogenic transformation. More significantly, adipogenic transformation remained evident after co‐culturing keloid fibroblasts with human scalp primitive HFs. Taken together, there is great potential for HFs to induce adipogenic reprogramming through BMP in scar therapy.

Subsequently, Hoerst et al.^60^ validated the critical role of BMP4 in myofibroblast adipogenic differentiation. Specifically, the α‐SMA phenotype of myofibroblasts was markedly reduced and did not induce adipogenic differentiation of hypertrophic scar or keloid fibroblasts. El‐Hattab et al.^61^ proposed that human adipocyte‐conditioned medium promotes fibroblast conversion to myofibroblast transformation. Therefore, more studies should be conducted to draw more definitive conclusions and more advances are required to understand the mechanisms underlying myofibroblast reprogramming.

The development of lineage tracing tools has greatly improved our ability to detect the dedifferentiation of myofibroblasts; however, it is well‐known that there is a degree of heterogeneity among different fibroblasts in vivo, both in terms of gene expression and cytokine secretion profiles.^62^ We can infer that there is also a subtle possibility of heterogeneity in myofibroblasts from different sources that may affect the propensity for myofibroblast plasticity. Such problems can be addressed by means of single‐cell sequencing and cluster analysis, which has been used to uncover the heterogeneity of other types of tissues and cells.

In contrast, myofibroblasts are defined as fibroblast‐like cells containing actin, myosin and other muscle proteins that are found in many tissues in response to injury and inflammation, thus leading to contractile behaviour that allows excessive deposition of collagen and secretion of fibrillar cytokines. No characterization and specific markers have been shown to characterize and distinguish myofibroblasts from fibroblasts, although there are significant differences between fibroblasts and myofibroblasts with respect to cell morphology, and levels of α‐SMA and collagens 1 and 3 expression. This remains a challenge with promising results unless markers of specificity are clearly identified.

## 6 CONCLUSION

Collectively, myofibroblasts play an important role in wound contraction and the occurrence and development of pathologic scars during wound healing. Depletion of myofibroblasts to reduce fibrosis is one of the strategies to treat pathologic scars. Recent studies involving myofibroblasts have only focused on two aspects (inhibition of myofibroblast synthesis and acceleration of myofibroblast apoptosis). The possibility of reprogramming of this terminally differentiated cell has been ignored for a long time. Fortunately, recent data suggest that myofibroblast reprogramming can be induced to become adipocytes. Then, adipocytes have a striking role in promoting wound healing and preventing scarring. Notably, HFs can alter the fate of myofibroblasts to become adipocytes through a signalling pathway dependent on BMP. A more detailed understanding of changes in myofibroblast gene expression, transcriptional regulators, signalling pathways and epigenetic regulators during reprogramming may pave the way for new strategies for scar prevention or treatment. More studies on myofibroblast reprogramming are eagerly awaited.

## AUTHOR CONTRIBUTIONS

**Ying‐Ying Teng:** Investigation (equal); writing – original draft (lead). **Ming‐Li Zou:** Investigation (equal); writing – original draft (equal). **Xiao‐Jin Zhou:** Data curation (equal). **Jun‐Jie Wu:** Visualization (lead). **Si‐Yu Liu:** Data curation (supporting). **Zheng‐Dong Yuan:** Visualization (supporting). **Yuan Jia:** Methodology (equal); project administration (equal). **Kai‐Wen Zhang:** Methodology (equal); project administration (equal). **Xia Li:** Writing – review and editing (equal). **Jun‐Xing Ye:** Writing – review and editing (equal). **Feng‐Lai Yuan:** Conceptualization (lead); writing – review and editing (equal).

## FUNDING INFORMATION

This work was supported by the Natural Science Foundation of China (81770876), Natural Science Foundation of Jiangsu Province (Grant BK20191141), Top Talent Support Program for young and middle‐aged people of Wuxi Health Committee (BJ2020044; BJ2020057; HB2020043) and Fundamental Research Funds of Health and Family Planning Commission of Wuxi (M202024).

## CONFLICT OF INTEREST

The authors declare that there are no conflicts of interest.

## Data Availability

Data sharing not applicable ‐ no new data generated, or the article describes entirely theoretical research.
